# Reviving the Dire Wolf? A Case Study in Welfare Ethics, Legal Gaps, and Ontological Ambiguity

**DOI:** 10.3390/ani15131839

**Published:** 2025-06-21

**Authors:** Alexandre Azevedo, Manuel Magalhães-Sant’Ana

**Affiliations:** 1 CIVG—Vasco da Gama Research Center, EUVG—Vasco da Gama University School, 3020-210 Coimbra, Portugal; 2 CIISA—Centro de Investigação Interdisciplinar em Sanidade Animal, Faculdade de Medicina Veterinária, Universidade de Lisboa, 1300-477 Lisbon, Portugal; mdsantana@fmv.ulisboa.pt; 3 Laboratório Associado para Ciência Animal e Veterinária (AL4AnimalS), 1300-477 Lisbon, Portugal

**Keywords:** de-extinction, animal welfare, genome editing, five domains model, animal ethics, biotechnology ethics, animal law, conservation, rewilding, wolves

## Abstract

The recent birth of genetically modified canids phenotypically resembling the extinct dire wolf (*Aenocyon dirus*) was hailed as a landmark in synthetic biology. Using genome editing and cloning, the biotech company Colossal Biosciences created three such animals from gray wolf cells, describing the project as an effort in “functional de-extinction”. This case raises significant questions regarding animal welfare, moral justification, and regulatory governance. We used the five domains model framework to assess the welfare risks for the engineered animals, the surrogate mothers used in reproduction, and other animals potentially affected by future reintroduction or escape scenarios. Ethical implications are examined through utilitarian, deontological, virtue, relational, and environmental ethics. Our analysis suggests that the project suffers from ontological ambiguity: it is unclear whether the animals created are resurrected species, hybrids, or novel organisms. While the current welfare of the engineered animals may be manageable, their long-term well-being, particularly under rewilding scenarios, is likely to be compromised. The moral arguments for reviving long-extinct species are weak, particularly in cases where extinction was not anthropogenic. Legally, the current EU frameworks lack the clarity and scope to classify, regulate, or protect genetically engineered extinct animals. We recommend that functional de-extinction involving sentient beings be approached with caution, supported by revised welfare tools and regulatory mechanisms.

## Simple Summary

Scientists have recently created animals that resemble the extinct dire wolf using genetic engineering and cloning techniques. These animals, born in 2024 and 2025, were produced by editing the DNA of modern wolves and implanting the embryos into surrogate dogs. The company behind this project claims that it could help bring back extinct species and support conservation. However, this technology raises serious questions about the welfare of the animals involved, the goals behind such efforts, and whether existing laws are able to protect these animals. This paper looks at the case of the engineered dire wolves to explore whether (a) the animals are likely to suffer, (b) it is ethically acceptable to create life forms based on long-extinct species, and (c) current regulations are prepared to deal with such cases. We found that these animals face long-term welfare risks, that the moral justification for recreating long-extinct species is weak, and that European legal frameworks are ineffective in addressing these emerging biotechnologies, particularly those involving de-extinction. We recommend caution and call for improved ethical and legal tools to guide future developments in this area.

## 1. Introduction

In 2013, a TEDx conference [1] brought de-extinction into the spotlight among the scientific community, leading to a polarized debate [2,3]. As back-breeding, somatic cell nuclear transfer and genetic engineering were being developed [4], increasingly successful attempts to “resurrect” or “create” animals were achieved. Examples include the cloning of sheep [5], the endangered mouflon (*Ovis orientalis musimon*) [6] and gaur (*Bos gaurus*) [7], the recently extinct Pyrenean ibex (*Capra pyrenaica pyrenaica*) [8], and cynomolgus monkeys (*Macaca fascilcularis*) [9]. More recently, genetic engineering has promised to boost the de-extinction toolbox by allowing researchers to edit the genome sequence of living animals to recover traits of extinct species [4]. While the leading proponents of the technology clarify that it can bring back traits, and not species [4,10], discussions have revolved around bringing back several extinct species, including (among others) the passenger pigeon (*Ectopistes migratorius*) [11], the thylacine (*Thylacinus cynocephalus*) [12], and the woolly mammoth (*Mammuthus primigenius*) [13]. These possibilities raised several questions regarding the ethics of de-extinction [3,11,14,15,16], the welfare of the animals involved [11,16,17], and the associated legal implications [3,16,18]. Among many who discussed its implications (e.g., [11,15,16,19,20]), Sherkow and Greely [3] discuss the major benefits and risks of de-extinction, proposing five categories for each. Objections include animal welfare concerns, health-related risks, environmental threats, political challenges, and moral objections. The proposed benefits of de-extinction have to do with scientific knowledge, technological advancement, environmental benefits, justice, and “wonder”.

Beyond the ethical debate around de-extinction, underlying discussions have focused on *what* the generated animals are, *where* they sit in the evolutionary tree, or *which* species should be selected for de-extinction. Ontogenetically, the question arises whether the gene-edited animals belong to (or are proxies for) the extinct species, or they belong to (or are hybrids/chimeras) of the surrogate species [4,18,21]. Phylogenetically, the question of species selection for de-extinction has been systematically addressed by conservationists [22,23], focusing mostly on a harm–benefit analysis. However, actual species choice might be subject to bias [24] and seems to revolve more around feasibility than necessity [13] (pp. 17–47).

Just recently, Colossal Biosciences achieved the first live births of animals genetically engineered to phenotypically resemble the extinct dire wolf (*Aenocyon dirus*)—a large carnivore that roamed North America until ~10,000 years ago [25]. News of this success includes prospects of Colossal using this technology to not only revive the dire wolf and other extinct species, but also to support conservation of extant species, e.g., by cloning red wolves (*Canis rufus*) or gene-editing resistance to cane toad toxins in the northern quoll (*Dasyurus hallucatus*). While the project represents a milestone in genetic engineering and synthetic biology, it raises serious concerns from an animal welfare perspective—particularly around the well-being of the engineered and surrogate animals, their long-term fate, and regulatory oversight. In this article, we aim to evaluate the scientific, ethical, and legal dimensions of engineered de-extinct animals, using the dire wolf as a case study. Specifically, we aim to address three research questions: (1) which animal welfare issues and risks arise from this endeavor? (2) should we create organisms modeled on long-extinct species? and (3) are current legal frameworks capable of regulating the welfare, breeding, use, and restoration of these animals?

## 2. Case Presentation

In late 2024 and early 2025, three genetically engineered wolf pups—Romulus, Remus, and Khaleesi—were reportedly created by Colossal Biosciences and were born at a secret wildlife facility in the United States of America [25]. These animals are portrayed in the news report as reconstructions of the dire wolf, which vanished approximately 10,000 years ago. According to the report, the animals display distinct morphological and behavioral traits consistent with their ancestors, including large size, powerful builds, wide heads, and solitary wild dispositions. The article goes on to quote Colossal Biosciences’ lead researcher: “Our mammoths and dire wolves are mammoths and dire wolves by that definition. They have the key traits that make that lineage of organisms distinct.” At six months of age, the pups are reported to weigh around 36 kg and are predicted to grow to about 68 kg and 1.8 m in length. Morphological differences from the gray wolves (*Canis lupus*), from whose cells they were created, include white coats, more powerful shoulders, wider heads, larger teeth and jaws, and more muscular legs. Behavioral traits reported in the article include avoidance behavior in relation to humans, early howling, and predatory behaviors such as stalking and chasing. Although the authors do not aim to define them as such, the animals produced from this research will be referred to throughout the paper as “dire wolves” for simplicity.

The technology reported to have created these pups involved the use of gene-editing techniques on gray wolf cells [25]. First, ancient DNA recovered from two dire wolf fossil specimens was sequenced, and key genetic differences between dire wolves and gray wolves were identified. Instead of inserting this DNA into gray wolf cells, researchers used it as a template to determine 20 specific edits across 14 gray wolf genes that would be necessary to recreate the physical and behavioral dire wolf traits. These edits were then made on isolated endothelial progenitor cells from gray wolf blood, and the nuclei of these cells were then transferred to denucleated dog ova. The engineered embryos were then implanted into surrogate dogs, who uneventfully carried them until birth by planned cesarean sections. The pups were reportedly placed with only one of the surrogate mothers but were removed for hand rearing after a few days because the mother became “too attentive—disrupting the pups’ regular sleeping and feeding schedules” [25]. The pups were bottle-fed until about eight weeks of age, and then weaned onto pureed meat, and later a whole meat diet. The pups live on a 2.000-acre (≅8 hectare) ecological preserve (though they are confined to a limited area within it) surrounded by a 3 m fence, with round-the-clock veterinary oversight. The article describes a naturalistic habitat with vegetation and wild animals, as well as natural dens, although no information on actual enclosure size or resources is presented.

Based on Kluger’s article in TIME [25], Colossal Biosciences frames the return of the dire wolf as a scientific novelty, and an initiative to serve ecological, technological, and even ethical goals. Kluger paraphrases Colossal’s public statements, saying that the company “claims that the same techniques it uses to summon back species from the dead could prevent existing but endangered animals from slipping into extinction themselves.” The article also quotes Colossal’s Chief Science Officer (CSO) Beth Shapiro, saying that “We are an evolutionary force at this point,” and that “We are deciding what the future of these species will be”. Further, summarizing the views expressed by Colossal’s executives, Kluger says they “frame the technology not just as a moral good, but a moral imperative—a way for humans, who have driven so many species to the brink of extinction, to get square with nature”. He supports this statement by quoting Beth Shapiro: “If we want a future that is both bionumerous and filled with people, we should be giving ourselves the opportunity to see what our big brains can do to reverse some of the bad things that we’ve done to the world already”. Colossal’s de-extinction webpage [26] mirrors these claims, defining functional de-extinction as the “process of generating an organism that both resembles and is genetically similar to an extinct species by resurrecting its lost lineage of core genes; engineering natural resistances; and enhancing adaptability that will allow it to thrive in today’s environment of climate change, dwindling resources, disease and human interference”.

Despite the recognition of the scientific and technological achievement, much of the responses to Colossal’s claim of de-extincting the dire wolf have focused on the scientific consensus that the animals are, in reality, genetically modified gray wolves and not genuine dire wolves [27,28,29]. Critics have also questioned the reasons for calling them dire wolves, and whether these results should have been publicized in the media before being published in scientific peer-reviewed journals [30]. However, as similar technological feats are expected to become increasingly common, further discussion is required regarding the welfare of the animals involved, and the ethical justifications for such procedures. Over the next sections, we use the dire wolf de-extinction case to discuss the welfare of the animals, the ethical justifications and concerns, and the potential regulatory void concerning the resulting animals.

## 3. Animal Welfare Science

To explore the welfare implications of Colossal Biosciences’ de-extinction of the dire wolf, we applied the most widespread model used for animal welfare assessments: the five domains model [31,32]. The five domains model explicitly acknowledges that welfare assessments are necessarily inferential, relying on observable circumstances and scientifically supported indicators rather than direct access to subjective experience. As such, the model permits—and indeed prescribes—the use of structured, evidence-based reasoning to evaluate potential affective states, making it fully applicable even in cases where the animals themselves are not directly accessible [33]. While inferential in nature—and therefore limited by the available information—this method enables the cautious, model-based projections of welfare risks grounded in reported facts and established scientific reasoning. The approach suggested to operationalize the model in forensic contexts [34] provides a targeted approach for identifying potential negative affective outcomes. This is particularly suited to the current case given its novelty and ethical ambiguities. As with any welfare assessment, context is critical. Therefore, for the purpose of this analysis, we set the working assumption that Colossal’s vision of “functional de-extinction” will materialize, implying that, at some point in the future, genetically modified wolves will be introduced into a natural ecosystem to restore an ecological function associated with their de-extinct phenotypic traits. Against this background, we attempt to infer the potential welfare outcomes across four categories of animals relevant to the project: the engineered dire wolves (Table 1), the surrogate mothers, the cell donor animals, and other animals potentially impacted by the application of this technology, future reintroduction, or escape scenarios.

### 3.1. Welfare of Engineered Wolves

Romulus, Remus, and Khaleesi are reported to receive a nutritionally appropriate diet, initially through surrogate nursing, then bottle-feeding, and then gradual weaning onto whole prey. Assuming this care continues, negative affective states due to nutrition are unlikely. However, if rewilding of wolves with dire wolf phenotypes occurred, appropriately sized prey would probably be unavailable amidst the current “herbivore defaunation crisis” [35]. To understand the implications of this absence, we need to revisit the cause of dire wolf extinction. According to paleontological evidence, the leading theory is that dire wolves were unable to survive the extinction of their large prey due to their large body size compared to competing predators [36,37]. Those predators were coyotes (*Canis latrans*), cougars (*Puma concolor*), bobcats (*Lynx rufus*) and gray wolves, which still exist today, in a context of increasing prey scarcity. Therefore, rewilded dire wolves would be expected to face even worse odds of surviving than their genetic ancestors, with a high risk of negative affective states of varying intensities and durations due to hunger and starvation.

Although the exact size of the enclosures is undisclosed, the environment where the genetically modified wolves are kept is depicted as physically spacious and is likely to exceed by far the space awarded to wolf species in zoological parks. However, it is nonetheless delimited by fences and lacks a prey base of large animals. No evidence of such issues can be derived from the report. Nevertheless, wide-ranging carnivores with itinerant lifestyles like wolves are particularly prone to captivity stress [38], making the welfare impacts of a limited environment likely in the long term. Weather extremes are reportedly dealt with by using natural dens. However, the functional phenotypic traits that were de-extinct in this research, including large body size and white coat, are typically related with cold climates. Hence, there is potential for a mismatch with current warmer environments, and the risk of thermal stress must be considered, if the traits are in fact those of a dire wolf.

Moreover, these animals were reportedly hand-reared, meaning they have been socialized to humans, rather than to adult conspecifics or a pack. They also lack contact with prey animals. As a result, they are likely to lack essential skills for sociability, hunting, and risk avoidance, rendering them unprepared and, admittedly, incapable of surviving independently in the wild. Considering the future prospects of rewilding genetically modified wolves, the concerns of environmental suitability increase, since they risk being exposed to competing predators, humans, temperature extremes, roads, and vehicles, among other environmental challenges. Arguably, the current wild animals are all subject to these environmental sources of welfare risks. However, they are currently the phenotypic result of selection within their environments, contrary to the genetically modified wolves.

The reported success of the program suggests positive health outcomes, at least in the short term. The wolves were born via planned cesarean sections and are reported to have been successfully reared and weaned, with no mortality reported [25]. However, the use of genome-editing technologies is associated with a degree of unpredictability in the long-term outcomes. Pleiotropic effects—where a single genetic modification can impact multiple unintended traits—can result in health issues that might only be apparent later in the wolves’ lives [39]. Large offspring syndrome is a known risk in these cases, leading to the increased rates of abortion, congenital malformations, and perinatal mortality [39]. Although the incidence of this problem has decreased with technological advancement [40], the fact that these pups were born by planned cesarean section suggests this risk was considered. While it does not seem to have resulted in any direct welfare impact on the pups, if the functional de-extinction of the dire wolf traits were to materialize through rewilding, the free-roaming wolves would need to breed naturally, increasing the risk of such occurrences. Another concern is the risk of physiological, immunological, or developmental dysfunction due to the risk of insertional mutations and ectopic transgene expression [39]. For example, pigs engineered to express higher levels of growth hormone unexpectedly expressed it in multiple organs, leading to an array of disorders, reduced fertility, and early mortality [39]. Another example is the editing of genes to produce erythropoietin in the mammary tissue of rabbits, which led to its unintended expression in several tissues, leading to an array of pathologies and premature deaths [41]. Almost twenty years later, current technologies are expected to have reduced these risks. However, a scarcity of reports in the literature is notable since then. Further, the type of management the wolf pups were subject to suggests some concern, at least related to birthing. Therefore, we can conclude that, in the domain of health, the risk of health-related welfare problems throughout the life of the pups is, at best, unknown, and high in the case of rewilded animals subject to natural breeding. Finally, it is difficult to predict whether these genetically modified wolves will be more resistant or susceptible to all the diseases circulating in the current ecosystems. Translocation-significant disease incursions are a significant risk to translocation projects, and are more likely to affect the introduced animals [42]. For example, even in the successful translocations of gray wolves, canine distemper virus and sarcoptic mange outbreaks have led to population declines [43]. If this is true for contemporary species, it is reasonable to consider that the risk will be even higher for phenotypes derived from long-extinct genomes.

Several welfare issues related to behavior can be inferred from Kluger’s chronicle [25]. The first is maternal separation and deprivation. Being removed from the surrogate mother at a few days of age can have implications in the welfare and development of the pups, resulting in behavioral abnormalities such as stereotypic behavior and changes in anxiety and stress responsiveness, and social behavior (reviewed in [41]). Evidence of the effects of human rearing includes conflicting results, with the increased incidence of behavioral abnormalities in most cases, but decreases in some [44]. Heightened fear and aggression and poor social and parenting skills are reported for human-reared animals, but in some cases, they exhibit less behavioral problems in captive zoo environments [44]. Romulus, Remus, and Khaleesi are likely to have experienced negative affective states associated with maternal separation, but human rearing might attenuate negative affective states (e.g., fear) related to their captive life with ubiquitous human presence. However, once considering the final goals of functional dire wolf de-extinction, the separation of pups for hand rearing would translate into a myriad of behavioral welfare impacts for the individuals and their offspring, ranging from the increased likelihood of wolf–human conflict, poor parenting, and social behavior. Even if they were raised by surrogate dogs, we can only conjecture about their natural behavioral repertoire. In addition to the risks of having gray wolves raised by surrogate dogs, we need to consider the possibility of behavioral phenotypes and needs associated with the engineered traits. Even if they were raised by their surrogate mother, it is highly unpredictable whether that would fulfill those needs. The second issue is related to the social context of these wolf pups. Although the article lacks detail, we can infer that the maximum group size for these animals is three individuals and that, at least for some periods, Khaleesi remains alone [25]. The pack size of dire wolves is unknown. However, large pack sizes are necessary for gray wolves hunting large prey, with optimal pack sizes of more than nine individuals required to efficiently capture a bison (*Bison bison*) [45]. Given that dire wolves evolved to prey on megafauna, it is reasonable to assume the pack size would be at least comparable to this size. Therefore, whether these pups are considered gray wolves or dire wolves, their small pack size subjects them to a high risk of limited opportunities for social bonding, exploration, intraspecific interactions, and reproduction. All of these can lead to frustration and behavioral stunting. Finally, the absence of large live prey constitutes another welfare issue for these powerful predators. The wolf pups are reported to express species-typical traits such as howling, stalking, and the cautious avoidance of humans, yet these behaviors lack appropriate outlets. Besides the limited pack structure that deprives them of opportunities for affiliative bonding, together with the lack of an adequate prey base, it deprives them of cooperative hunting. Therefore, the lack of an appropriate prey base restricts the expression of highly motivated and evolved predatory drives, creating a risk for long-term frustration. The absence of large-sized prey is likely to hold true in rewilded individuals.

The fifth domain of the model reflects the cumulative affective outcomes of the animals’ internal state, as inferred from their nutrition, environment, health, and behavior. Several domains indicate the risks of negative affective states for de-extinct dire wolves, particularly as they mature. The absence of suitable prey in modern ecosystems represents a risk of chronic undernourishment and frustration of unfulfilled predatory drives. Depending on the severity and duration of the mismatch between motivation and environmental opportunities, this could potentially result in states of agitation, anxiety, or lethargy. Environmental factors such as fences, climatic conditions, or habitat types mismatched with the phenotype limit the animals to express their evolved behaviors. While this is not currently reported to induce distress, over time, it may lead to boredom, frustration, or a narrow range of behavioral and affective expression. However, unpredictable pleiotropic effects or specific disease susceptibilities can manifest later in life through chronic illness, pain, or weakness and the associated affective states of discomfort, depression, or distress. These effects could be particularly relevant in semi-wild or wild settings. In the behavioral domain, early maternal separation can lead to impaired emotional regulation, increased stress reactivity, and abnormal social behavior, but could also buffer the negative effects of captivity and human proximity. The current group size also provides a socially impoverished environment, particularly for a species that requires large packs for cooperative hunting and affiliative behavior. A small group size increases the risk of loneliness, social frustration, and behavioral stunting. Finally, the absence of opportunities for cooperative hunting of large prey suggests these wolves will be unable to fulfill high-motivation predatory behaviors that are not only innate but intrinsically rewarding. The inability to express these behaviors may cause a chronic affective state of frustration. Altogether, the cumulative result of the mental domain indicates a mild current welfare compromise, but a high risk of severe welfare compromise in the long term, particularly in scenarios involving rewilding or natural reproduction. Most risks emerge from the limitations of the artificial environment and rearing and the mismatch between the engineered traits evolved for now-vanished ecological conditions and current ecosystems.

**Table 1:** Inferred welfare assessment for the engineered wolves.

Domain	Descriptors and Indicators [25]	Inferred Affective States	Welfare Risks
Nutrition	Adequate, species-appropriate diet in terms of nutrition; weaning at 8 weeks; transition from surrogate nursing to whole-prey-like feeding	None	Low risk if kept captive and provision remains stable High risk if rewilded, and limited access to suitable prey [35]
Environment	Enclosed but large; lacks environmental variability; lacks prey base related to de-extinct traits	Mild frustration Low stimulation	High risk of under-stimulation if kept captive [38] Low risk if rewilded
Health	No current signs of illness; proactive monitoring and veterinary care; unknown long-term outcomes of gene editing (e.g., pleiotropic effects and disease vulnerability)	None	Unknown but potentially severe risk of delayed onset issues due to pleiotropy, organ dysfunction, premature aging, or disease susceptibility [39]
Behavior	Maternal deprivation; hand rearing and risk of imprinting; only 3 individuals, potentially undergoing isolation even if for short periods; limited opportunities for social bonding, exploration, or reproduction; no hunting of large live prey; frequent contact with humans and expression of avoidance behavior	Maternal deprivation Thwarted predatory drive Social isolation Inhibited agency Fear (of humans)	High risk of frustration and anxiety if natural behaviors remain unexpressed Low risk if rewilded in appropriately sized groups of parent-reared individual
Mental State	Express wild traits (howling, stalking), but without appropriate outlets or conspecific feedback	Latent loneliness Cognitive under-stimulation	High risk in captivity due to unmet cognitive, environmental, and social needs Unknown risk over time due to pleiotropic effects and disease susceptibility

### 3.2. Welfare of Surrogate Animals

Although no gestational problem or miscarriage is reported to have occurred, the dire wolf de-extinction required surrogate dogs that underwent embryo transfer and cesarean sections [25]. There is no reason to assume that these surrogate dogs experienced relevant welfare issues associated with nutrition or the environment. However, the use of surrogate animals raises its own set of welfare concerns associated with the domains of health and behavior. Regarding health, embryo transfer procedures typically involve hormonal monitoring and laparotomy under anesthesia [46]. Extensive evidence on the anesthetic risk in dogs shows that anesthesia has a (albeit low) risk of death, even in healthy dogs [47]. Conversely, the risk of poor-quality anesthetic recoveries may be higher in healthy dogs, potentially including behaviors such as restlessness, vocalization, agitation, aggression, and convulsions [48]. Additionally, laparotomy is associated with an increase in cortisol [49], tissue damage, pain [50], and surgical complications, even if minimally invasive methods are used [51]. In clinical practice, planned pre-parturient cesarean sections can be considered a safe alternative to natural delivery (eutocia) in cases where a risk of emergency cesarean section is identified [52]. However, like laparotomies, cesarean sections are associated with post-operative pain and carry an anesthetic and surgical risk for surrogates [53]. Additionally, cesarean sections are likely to be more stressful for the bitch, when compared with eutocia [54]. Most of the health and behavioral impacts derived from embryo transfer and planned cesarean sections can be managed to levels generally accepted for bitches in daily clinical practice. Hence, the discussion on whether these animals should be subjected to these procedures is mostly an ethical one. However, a separate concern within the behavioral domain is the effects of maternal separation on the dam. In rats, repeated maternal separation in the post-parturient period has been shown to lead to depression or anxiety-like behavior [55]. Several species of animals exhibit behaviors like vocalization, locomotion, searching behavior, altered feeding and sleep patterns, reduced play behavior, and increases in corticosteroids, heart rate, and body temperature (reviewed in [56]). Finally, all the above issues are reflected on the mental domain of surrogate dams in dire wolf functional de-extinction. Welfare issues associated with anesthetic and surgical procedures can be managed to reduce their effects on the affective states. However, it is impossible to eliminate pain, fear, and discomfort completely from these procedures. The behavioral signs of distress in dams subject to the early separation of pups are associated with the neural activation of the amygdala [55], which plays a central role in processing emotions such as fear and anxiety. This indicates that these behaviors signal negative affective states and distress, which can last for several days [56]. Therefore, the burden of reproductive manipulation on the surrogate dams cannot be ignored, especially if they are used for more than one gestation or if the process is scaled to produce more dire wolves for rewilding.

### 3.3. Welfare of Donor Animals

In addition to surrogate mothers, Colossal’s research required blood cells from gray wolves. Although detailed information about the sourcing of the gray wolf blood is not provided, it is reasonable to assume that these samples can be obtained opportunistically from animals undergoing routine handling—such as veterinary check-ups, conservation interventions, or zoo-based procedures. In this context, blood collection is a standard and low-impact procedure.

However, minor welfare concerns remain worth acknowledging. Physical or chemical restraint for venipuncture can induce transient stress, particularly in wild animals. While these impacts are generally transient and short-lived, repeated handling and sampling, particularly in wild or non-habituated animals, could lead to significant welfare impacts that could become relevant if larger numbers of dire wolves were produced for release into the wild. Furthermore, if other forms of tissue sampling, such as skin biopsies, are involved in creating engineered animals, these would represent a higher welfare burden. As the scale of de-extinction efforts grows, these considerations become more pressing and require greater ethical scrutiny.

### 3.4. Welfare Risks to Other Animals

The welfare risks to other animals need to be examined considering the end goal that justifies the functional de-extinction research. Recovering extinct phenotypical traits and their functions in the ecosystems implies rewilding de-extinct specimens in a natural ecosystem. Welfare outcomes on individual, population, or ecosystem levels are difficult to predict, but some lessons can be drawn from human interventions in the past. Analogous discussions on biological invasions [57] and assisted colonization [58,59] have highlighted this unpredictability, and provide many examples of unintended negative outcomes. In the domain of nutrition, reintroducing dire wolves into contemporary ecosystems risks disrupting established populations that have evolved without such an apex predator [57]. This could lead to changes in foraging behavior and nutritional intake of herbivores, and on prey availability for competing carnivores. Apex predator reintroductions can also affect the environment through cascading effects resulting from changes in herbivore populations [60]. Impacts on the health of native animals are also unpredictable. Although a well-managed program would reduce the risk of introducing diseases through dire wolf rewilding, there is an unknown risk of disturbing disease dynamics in the ecosystems due to potential vulnerability or resistance of the introduced animals to endemic diseases, and to the effects of the introduction on the disease dynamics in other species. Regarding behavior, introduced predators can influence the behavior of native species of predators through intraguild competition [61] and prey species through the ecology of fear [62]. The diversity and unpredictability of the potential impacts of rewilding de-extinct dire wolves makes any attempt to infer mental states of individual animals from the ecosystem futile. However, when discussing the same concerns in the context of assisted colonization, experts estimated risks on native biota to be of high importance, with the majority of them estimating specific risks of disease transmission and competition or displacement of native species [58]. Therefore, the potential welfare impacts derived from these risks merit careful consideration.

## 4. Ethical Analysis

Extinction is a fundamental part of evolutionary history; indeed, over 99% of all species that have ever lived are now extinct. However, the context in which extinction occurs matters deeply. Today, we are facing what scientists describe as the Sixth Mass Extinction [63,64,65], a crisis not driven by asteroid impact or volcanic eruptions, but by human activity—namely habitat destruction, pollution, climate change, and the spread of invasive species. In cases where human influence is the primary cause, it can be argued that we have a moral responsibility to intervene, mitigate, or even reverse the damage. This logic underpins efforts to conserve endangered species and, increasingly, support some forms of de-extinction. De-extinction can be defensible in cases of recently lost species, particularly those with surviving ecological niches and where restoration may support broader conservation goals. In contrast, reviving long-extinct species such as dire wolves, who vanished more than 10,000 years ago [36], is a fundamentally different proposition—biologically, ecologically, and ethically. Several ethical theories offer distinct perspectives on the de-extinction of the dire wolf. In this section, we will explore the ethics of dire wolf de-extinction using a normative approach, through the lens of five chief ethical frameworks: utilitarianism, deontology, virtue ethics, relationism, and ecocentric environmental ethics (Table 2). For the sake of clarity and conciseness, our analysis will focus specifically on the engineered wolves.

### 4.1. Utilitarianism

Utilitarianism approaches the issue by weighing the potential suffering experienced by surrogate animals and gene-edited wolves against the possible long-term benefits, such as advancing conservation science and biodiversity restoration. This rationale is behind most of the claims from Colossal Biosciences’ CSO, including that “these are the luckiest animals ever” [25]. Under utilitarianism, de-extinction may be considered ethically justifiable, provided the expected long-term benefits, such as restoring ecosystems, advancing scientific knowledge, recreational value, or correcting past harms, are greater than the harms involved (including financial risks, health concerns, environmental hazards, and animal suffering [14]). However, utilitarianism would demand the strong evidence of positive utility, and in the case of long-extinct species like the dire wolf, benefits remain speculative. In the words of Shlomo Cohen, “Utilitarianism never *truly* delivers on its promise, since a complete evaluation of consequences is virtually never possible in real-world open systems” [14] (p. 175).

Adam Shriver [68] provides a cautionary view from within a utilitarian tradition. He acknowledges that genetic engineering can reduce suffering or serve biomedical goals, but stresses that such interventions must not institutionalize or normalize harm to animals for marginal human benefit. Shriver highlights the importance of evaluating whether these procedures genuinely improve animal well-being or simply serve vested human interests under a scientific or conservationist rhetoric. Shriver invokes the non-identity problem to question simplistic justifications for genetically creating animals who will likely suffer. Applied to de-extinction, his perspective would demand not just hypothetical benefits but verifiable improvements in animal welfare and ecological impact. If evidence emerges that the procedure causes significant suffering (to surrogates or engineered wolves) or yields minimal ecological utility, it could be ethically indefensible, even under a utilitarian framework.

### 4.2. Deontology

Deontology (when applied to animals) is centered on our moral duties towards the intrinsic value of sentient beings. Colossal Biosciences’ CSO frames de-extinction as a “moral imperative”, asserting that humans “are deciding what the future of these species will be” and should use their capabilities to reverse past ecological harms. This perspective raises significant ethical questions, particularly when examined through deontological ethics.

Some proponents of de-extinction argue, from a duty to repair, that we are morally obligated to restore species we have driven to extinction. Paleontologist Michael Archer, for example, has argued that, if humans were clearly responsible for species’ extinction, then, there is a moral imperative to try to bring them back, if it is technically possible. He cites the thylacine and the gastric-brooding frog (species made extinct by human actions, and the last representatives of a now lost family line) and defends their revival as an act of moral restitution [69]. This logic, however, does not apply to the dire wolf. The extinction of *Aenocyon dirus* occurred approximately 10,000 years ago, long before any credible evidence of human causation [36]. As such, it could be argued that no clear moral debt is owed to this species.

On the contrary, deontology opposes animal instrumentalization and would therefore likely condemn the dire wolf project. From this viewpoint, it is deemed unethical to create life forms through invasive and artificial means merely as a means to human ends, such as corporate promotion, scientific advancement, or symbolic restitution. Even if the intended outcomes appear beneficial, the act of creating sentient beings through invasive means primarily for environmental restoration raises serious aprioristic ethical concerns. While proponents may argue that the ultimate goal is ecological rather than anthropocentric, from a deontological standpoint, this does not override the duty to treat animals as ends in themselves—even in the service of environmental ideals. In Justice for Animals [70], Martha Nussbaum’s reinforces this critique by insisting that justice requires more than minimizing harm; it demands that all sentient beings be given the opportunity to flourish according to their “central capabilities”. Nussbaum’s “Capabilities Approach” argues that sentient animals have “entitlements” (i.e., basic moral claims) grounded in their ability to live a flourishing life according to their species-specific nature, and not merely as a result of their usefulness to human purposes [71]. De-extinct animals, created through genetic engineering and cloning, are likely to face compromised behavioral, social, and ecological opportunities. In Nussbaum’s view, bringing animals into existence without being able—or willing—to support their natural needs is not an act of care or progress, but a new form of injustice. Moreover, this critique becomes even more pressing in light of ontological ambiguity. If it is unclear whether the genetically modified canids are revived dire wolves, modified gray wolves, or entirely new organisms, it becomes difficult to determine what kind of moral subject they are. This uncertainty hinders our ability to apply consistent obligations and regulations, since the moral status of a being often depends on its species identity or natural history.

### 4.3. Virtue Ethics

Virtue ethics shifts the lens from consequences and rights to the moral character of the people and institutions engaging in de-extinction. It asks what it says about us if we choose to create these animals. Virtue ethics would be cautious or critical, depending on the motivations and character traits demonstrated by those undertaking the research. If the project is driven by feelings of ambition, economic gain, or showmanship, it is deemed morally dubious. If motivated by phronesis (practical wisdom), justice, and compassion, it might be considered more favorably.

Rosalind Hursthouse [72] argues that practices that involve routinely harming or instrumentalizing animals promote vicious behaviors in their practitioners. Hursthouse’s work invites us to ask not only what we are doing, but who we are becoming in the process. Applied to de-extinction, this means that, even if the engineered animals end up having good lives, the act of creating life forms for human purposes—especially under uncertain welfare and environmental benefits—may reflect a flawed moral character. This is particularly evident in the rhetoric surrounding de-extinction: while proponents claim that revived species will inspire awe and wonder, the source of that awe is no longer nature’s wild majesty, but our own technological prowess [15]. The CSO’s statements can be interpreted as displaying hubris (“we are an evolutionary force”) and technological overconfidence, which denote the arrogance and recklessness of “playing god” [14,16]. From a virtue ethics perspective, such self-congratulatory admiration may denote a moral failure to respect the natural world as nothing more than a platform for human achievement.

De-extinction initiatives have potential to provide insights into evolution, behavior, and comparative genomics and to drive technological advancements [3] that can be fed back into human healthcare via gene-editing innovation [73]. This can underpin a justification under virtue ethics, when the outcomes are pursued for practical wisdom or a benevolent purpose. However, this pursuit should be conducted in ways that minimize the instrumentalization and suffering of animals. A scientist committed to the virtues of prudence, humility, and care would likely approach such a project with skepticism, if not outright disapproval; not because it’s inherently wrong, but because it lacks moral proportion and restraint. One way to embody virtues such as compassion and moral restraint would be to prioritize non-sentient organisms or microbial models in the early stages of research, or at least domestic species with well-established needs and husbandry standards to reduce harm, where possible, and signal moral seriousness about the animals involved.

### 4.4. Relational Ethics

Relational ethics, based on emotional connections, may be invoked to support these de-extinction efforts. The justification for de-extinction in this case appears to be at least partially tied to a sense of wonder [3], public awe, and species charisma—what some call the “existence value” of iconic animals [74], i.e., the value people place on simply knowing that a species exists, even if they never see or interact with it. It is frequently referred for iconic or charismatic species—such as wolves, elephants, or dolphins—that carry symbolic and emotional significance. However, this approach is not without critique. It risks leading to a “Disneyfication” of nature, where ethical decisions are shaped more by cultural narratives and symbolic appeal than by the actual interests and needs of the animals involved [67]. The naming of the pups—Romulus, Remus, and Khaleesi—raises further concerns: is the appropriation of the Game of Thrones imagery [75] part of a strategically constructed public narrative that risks obscuring welfare and ethical considerations? Naming and classification are not neutral acts. By invoking the dire wolf identity, Colossal taps into the political and emotional capital associated with charismatic megafauna. This strategic labeling influences public perception, funding prospects, and even legal categorization, and risks reinforcing legitimacy through narrative and obscuring scientific and ethical ambiguities. Relational ethics involves our responsibility to develop meaningful interactions with other beings, including trust and care. Since no prior human–animal relationship existed with the engineered wolf, and no natural bonds can be assumed, this framework would question whether we can meaningfully care for or understand the needs of these animals in the future. Without an established relational history, or the ability to ensure their well-being, responsible care remains speculative. In the dire wolf case, relational ethics must not be reduced to sentimentality; it must remain grounded in responsibility, pragmatism, and the capacity to genuinely meet the animals’ needs.

### 4.5. Environmental Ethics

Environmental ethics includes a myriad of ethical frameworks. To explore the present case study, we will focus on ecocentric environmental views, which assign intrinsic value to species and ecosystems beyond human utility. Ecocentrism encourages us to preserve natural processes and respect the limits of human intervention, particularly concerning long-extinct animals like the dire wolf. Jessie Beier argues that de-extinction projects such as those promoted by Colossal Biosciences exemplify a modern form of dominion—a belief in humanity’s rightful authority to reconfigure life itself [76]. Rather than disrupting anthropocentrism, these efforts often reaffirm it, framing extinction as a technological problem solvable by human ingenuity, and turning humans into both the cause of and the solution to ecological collapse. From this ecofeminist perspective, the language used by Colossal Biosciences’ CSO implies a position of dominion over nature, one that reflects a patriarchal, capitalist, and technocratic worldview, instead of demonstrating stewardship and reverence. Attempting to recreate an extinct apex predator for symbolic, scientific, or ecological engineering purposes reinforces the notion that nature is a tool for human ambition, rather than a system with its own intrinsic value.

Aldo Leopold’s Land Ethic [77] offers an equally critical perspective. He famously said that “a thing is right when it tends to preserve the integrity, stability, and beauty of the biotic community. It is wrong when it tends otherwise” (pp. 224–225). From this perspective, the dire wolf’s extinction was part of a natural ecological shift, not a moral wrong demanding repair. Since its historical ecosystem no longer exists, attempting to reintroduce a proxy species serves no restorative function. What we create now is not a recovered predator with a gene pool carved by thousands of years of natural selection, but a novel organism constructed from gray wolf DNA and modern approximations. This is not restoration; it is reinvention, and potentially one that destabilizes rather than strengthens the biotic community. In this light, de-extinction becomes an act of ecological vanity, violating the humility and restraint Leopold held as central to land ethics. Such initiatives conflict with ecocentric values that call for restraint, humility, and respect for the autonomy of natural systems.

## 5. Legal Dimensions

Legal frameworks are necessary to establish limits and safeguards to protect animals and public health, but they often struggle to accommodate novel entities like genetically modified animals [78]. To illustrate the difficulties in addressing the case of de-extinct dire wolves, we analyze the case under the European Union’s (EU) regulatory frameworks (Table 3). This approach was selected for the legal analysis for three reasons: (1) the EU maintains one of the most comprehensive and precautionary legislative frameworks worldwide regarding animal welfare, genetic engineering, and environmental protection [79]; (2) ethical dilemmas raised by synthetic and genetically modified organisms are most pressing in jurisdictions where the legal recognition of animal sentience and biodiversity obligations is robust, with the EU providing an advanced case study in this regard; and (3) given the authors’ positioning within the EU, this focus enables both professional familiarity and an delimited scope. Comparative legal analysis (e.g., US, UK, or international law) offers valuable contrasts, but falls beyond the scope of this conceptual case study.

Although the European Union has no legislation specifically addressing de-extinct or synthetic animals, several legal instruments may apply indirectly. In the present case, Romulus, Remus, and Khaleesi would be classified as research animals and therefore fall under the scope of Directive 2010/63/EU. As a result, all procedures involving cloning, embryo implantation, and postnatal care would require ethical review, harm–benefit analysis, welfare justification, and minimization of suffering through the process [80].

However, if created for conservation, rewilding, or display, other legal frameworks would become applicable. A key challenge is discerning which frameworks apply: are these animals best understood as genetically modified organisms, wild fauna, invasive alien species, or synthetic hybrids? Depending on the applicable regulations, the legal classification of the animals can be based on their biological characteristics (e.g., taxon), on the purpose for which they are used (e.g., farming, companionship, research), or on the context in which they are kept (e.g., in their native ecosystems, in non-native ecosystems, or confined in zoos).

The initial and overarching classification is as a sentient animal under the Treaty of the functioning of the European Union [81] which affords some protection in terms of how these animals must be treated. Under Directive 2001/18/EC [82], on the deliberate release into the environment of genetically modified organisms (GMO), an organism is considered a GMO if its genetic material has been altered in a way not occurring naturally by mating and/or natural recombination. This classification would likely apply to these animals. In cases involving rewilding, the release of gene-edited grey wolves would trigger requirements for environmental risk assessment, containment protocols, and possibly restrictions on breeding or transport, to prevent unintended ecological disruption and ensure regulatory compliance.

Another possibility is their classification as gray wolves (*Canis lupus*), albeit genetically modified. However, it remains unclear whether rewilded animals would be covered by the protection of regulations targeting wild wolves. The habitats Directive (92/43/EEC [83]) lists *Canis lupus* as a species of community interest for conservation and protects specific populations in their natural range, but whether modified individuals released into those ranges would fall under this protection is unresolved. Additionally, this scenario highlights another gap in regulation: the protection of native wolves and other species in the intervened ecosystems. One of the potential benefits of de-extinction is to challenge the scope of human intervention in nature and force critical discussions on the adequacy of current bioethics and technological governance frameworks [84,85]. The engineered wolves are neither fully wild nor fully synthetic, prompting the need for a revision of legal and normative categories. This gap could be partially resolved by their classification as invasive alien species if ecological impacts are documented [86]. However, this approach only offers a post hoc solution to potential harm, and such a classification is difficult to reconcile with the end goals of functional rewilding or ecosystem restoration that legitimize the creation of these wolves in the first place.

In other cases, if dire wolf specimens end up being kept as “exotic” pets or display animals at zoos or wildlife parks, they would fall under the protection of national companion animal protection laws (no single EU framework exists) or Council Directive 1999/22/EC [87], respectively. These regulations include specific determinations to protect the welfare of companion animals or zoo animals. However, the perspective of de-extinct dire wolves being kept as pets or on display in zoos raises an entirely distinct group of ethical questions that are beyond the scope of this work.

Finally, if considered as new species of dire wolf, or of the same species of extinct dire wolves, they could be classified as synthetic or novel organisms, or as a historically extinct species. As Okuno [88] highlights when discussing the case of mammoth de-extinction, there is a lack of regulatory frameworks capable of addressing the trade, protection, transboundary movement, and intellectual property of these animals.

The absence of a dedicated legal framework for de-extinct animals leaves unanswered questions about the protection of animals, public health, and natural ecosystems. This legal vacuum complicates governance and decision making and may create opportunities for abusive and illegitimate use of the technologies and animals involved.

**Table 3:** Dire wolf de-extinction under EU regulatory frameworks.

Classification	Legal Framework (EU)	Defining Criteria	Fit for Engineered Wolves?	Regulatory Implications
Wildlife (*Canis lupus*)	Habitats Directive (92/43/EEC); CITES	Species listed in Annex IV of Directive 92/43/EEC are strictly protected; includes natural range and habitats (Art. 12)	Ambiguous: engineered from gray wolf, but not naturally occurring	May not qualify for protection as “natural population”; reintroduction could violate habitat integrity rules
Domesticated animal	Animal Welfare Directive (98/58/EC)	Applies to animals bred or kept for farming purposes (Art. 1)	No: not socially adapted or selectively bred for domesticated traits	Likely excluded from pet/livestock regulations; unsuitable for companion animal welfare laws
Genetically modified organism GMO)	Directive 2001/18/EC (Deliberate Release of GMOs); Regulation 1829/2003	Organism whose genetic material has been altered in a way that does not occur naturally by mating and/or natural recombination (Art. 2(2))	Yes: genetically engineered animal to express extinct traits	Triggers strict risk assessment, notification, labeling, traceability, and post-release monitoring requirements; no regulation on welfare
Research animal	Directive 2010/63/EU	Applies to live non-human vertebrate animals used in procedures likely to cause pain, suffering, distress, or lasting harm (Art. 1)	Yes, while used in research; no, if bred for conservation or display	Requires ethical review, licensing, 3Rs’ compliance (Replacement, Reduction, Refinement); welfare strictly regulated
Synthetic or novel organism	No direct coverage; emerging policy gap	Organisms with no natural counterpart; synthetic genome traits	Arguably yes: traits engineered from extinct species not found in any current population; however, could also be considered gray wolves	Legal vacuum; requires new framework for oversight of classification, welfare, breeding, and environmental release
Zoo animal	Council Directive 1999/22/EC; national zoo licensing regulations	Applies to animals kept for public display in zoos or collections (Art. 2)	Possibly, if housed in regulated zoo settings	Subject to enclosure standards, vet care, species-specific needs, and welfare standards; does not resolve classification in law more broadly
Invasive alien species	Regulation (EU) No 1143/2014	Non-native species whose introduction or spread has been found to threaten or adversely impact biodiversity and related ecosystem services (Art. 3(2))	Ambiguous: genetically close to native wolves but not existing in wild populations	If considered distinct, may require risk assessments before release; restrictions on breeding or movement; would depend on their ecological impact
Companion animal (pet)	No single EU directive; national laws; Council of Europe Convention for the Protection of Pet Animals (1987)	Animals kept for companionship; Council of Europe Convention ETS 125 (Art. 1–3): sets welfare norms for breeding, housing, and use	Possibly, if socialized and privately owned	National pet laws would apply; engineered wild traits may conflict with exotic or hybrid species ownership rules
Sentient animal	Treaty on the Functioning of the EU (Treaty of Lisbon)	“In formulating and implementing policies… the Union and Member States shall pay full regard to the welfare requirements of animals as sentient beings”	Yes: principle applies to all vertebrate animals, including novel biotechnological forms	Provides constitutional basis for precaution, ethical oversight, and policy development even in legal gaps

## 6. Discussion

The de-extinction of the dire wolf via genetic engineering presents unprecedented challenges in animal welfare science, ethics, and legal policy. While Colossal’s methods are technologically impressive, the resulting animals risk living socially and ecologically constrained lives. Ethical justifications remain conflicted—balanced between poorly founded conservation goals and animal instrumentalization. Legally, the European Union lacks targeted regulation for such animals, risking welfare blind spots and ambiguity.

A central issue in this case is the clear definition of the end goals behind de-extinction. Two assumptions underpin the concept of functional de-extinction: first, that the traits that are being de-extinct are necessary and of positive conservation value; and second, that rewilding animals with those traits is ecologically necessary and feasible. Neither assumption is convincingly met in this case. The ongoing decline of herbivorous megafauna does not justify the ecological need for larger carnivores, with powerful jaws and white coats. Nor does it seem plausible to claim that current ecosystems need rewilded populations of such animals, especially when ecological functions could be fulfilled by native species.

This leads to a deeper ontological and ethical question: has Colossal truly revived the dire wolf or merely engineered gray wolves to exhibit specific phenotypes? The answer to this question is central to the discussion because it influences the perceived legitimacy of the research. The likelihood of funding, as well as social and political acceptance, would likely differ between the genuine de-extinction of a charismatic species and the phenotypic enhancement of a current species through genetic modification. Nelson and O’Riordan [89] argue that such projects “leverage conservation narratives to legitimize biotechnological advancements”. In this case, framing the project as dire wolf de-extinction—rather than gray wolf modification—and combined with the weak evidence for the need of de-extinct traits, supports the interpretation that a conservation narrative is being used to secure public support and funding rather than address genuine ecological needs. If restoration was the true aim, genetically enhanced or selectively bred gray wolves could likely address the need. Instead, Colossal appears to be pursuing a form of technological solutionism, where justifications are sought to justify the technological pursuit, rather than developing solutions to address established ecological or ethical needs.

This case exemplifies what may be termed a situation of ontological ambiguity—a concept referring to the difficulty or impossibility of clearly defining what kind of entity a genetically engineered organism actually is. Ontological ambiguity arises when entities defy conventional biological, ethical, or legal categories. The engineered animals in this case resemble dire wolves phenotypically but are genetically modified gray wolves. Are they resurrected species, hybrids, proxies, or something entirely new? Further complicating this ambiguity is the limited scope of the genetic intervention. Only 14 genes are reported to have been modified in this first iteration of the project, which illustrates the technical constraints limiting de-extinction to resemblance rather than genetic continuity. Additionally, this ontological ambiguity is not static. In cases where these animals are rewilded and breed, their biological identity may continue to diverge from any original archetype, defying any tentative categories we might apply and raising further questions on how to define and govern such organisms. Although, at first glance, the classification of such animals might be seen as a straightforward scientific endeavor, it is an extension of an ongoing philosophical discussion on the fluid concept of species [90]. This ambiguity has profound implications on our ability to assess the welfare and moral status of these animals, and to determine under which legal classifications and frameworks they are covered.

De-extinction projects, in general, might be justified by conservation needs and help restore ecosystem processes [91]. In the current case, the absence of concrete needs or restoration plans with timelines and locations (even if only speculative) raises questions regarding practical implementation, and whether rewilding could be part of a promotional rhetoric to legitimize the technological pursuit. Economic and recreational benefits are very likely to derive from this initiative, through what has been termed awe, wonder, or coolness [3,15]. The well-known Jurassic Park saga is an example of the recreational potential of this concept, particularly when applied to large, charismatic animals perceived as dangerous. Additionally, the dire wolf has recently entered the spotlight due to the successful Game of Thrones series. Besides recreational value, this can lead to other benefits, including the ability to increase awareness and public support for conservation and to raise substantial amounts of resources [91,92]. Under a utilitarianism framework, these benefits need to be carefully assessed for balance and proportionality with the suffering of the animals involved and the potential harms that are currently unknown.

No single ethical framework can adequately address the complexities of reviving long-extinct species. It challenges assumptions about species identity, naturalness, and the moral relevance of evolutionary lineage. A blended approach seems most appropriate: utilitarianism offers a structure for assessing outcomes, deontology sets necessary moral limits, virtue ethics ensures that our motivations remain aligned with moral integrity, and environmental ethics ensures caution when intervening in ecosystems that have long since adapted to the absence of a species. The fact that most perspectives align in advising caution, while only a consequentialist theory presents arguments in favor of the decision, mostly based on speculative outcomes, suggests that moving forward with this research might not be the best decision.

Some theorists, however, adopt a more permissive stance, which might be described as moral pragmatism. Philosopher Ronald Sandler argues that deep de-extinction (i.e., the de-extinction of long-extinct species) is neither intrinsically problematic nor morally obligatory [16]. In his view, deep de-extinction does not serve restorative justice, since species and ecosystems are not moral patients capable of being wronged in the first place. Instead, he argues that deep de-extinction should be treated as a luxury, a techno-scientific pursuit that is acceptable if it does not interfere with ethically important goals like conserving extant species, promoting animal welfare, or preserving ecological integrity [16]. This stance tolerates such projects without endorsing them as moral imperatives, placing the moral weight not on the act of de-extinction itself, but on its broader ethical context and opportunity costs.

The ontological ambiguity also complicates the regulatory analysis. As with other novel biotechnological entities—such as human–animal chimeras or synthetic organisms—de-extinct animals like Romulus, Remus, and Khaleesi do not fit neatly into established ontological legal categories [85]. This disrupts normative frameworks, which are often built around clear boundaries: wild vs. domestic, natural vs. artifactual, and extinct vs. extant. In this context, ontological ambiguity is not merely a philosophical curiosity. It is a central ethical and legal obstacle to the responsible governance of de-extinction technologies [93]. Yet this very challenge may present an opportunity, since de-extinction efforts such as the dire wolf project can push for the development of new legal instruments that can address the regulatory gaps created by these emerging biotechnologies [84,85].

## 7. Conclusions

Considering the complex ethical welfare and legal challenges posed by the engineered dire wolves, we argue for a precautionary stance toward de-extinction. Moreover, we argue that the de-extinction of long-extinct species to recover specific phenotypic traits should only be performed when a clear need is identified and a reasonable expectation of success in restoring the ecological function exists. While technological advances have enabled the recovery of phenotypic traits from extinct species, they have also outpaced the frameworks needed to ethically and legally regulate the beings they produce. The ontological ambiguity of these animals—neither the long-extinct species nor the entirely new ones—complicates how we assess their needs, moral status, and roles within ecosystems. To responsibly guide future developments, we recommend the expansion of welfare assessment frameworks to include sentient GMOs and synthetic organisms, the creation of new legal classifications to avoid jurisdictional gaps, and the establishment of interdisciplinary ethical review boards equipped to address the challenges of biotechnological innovations. Until such tools are in place, we recommend restraint in the applications of de-extinction technologies involving sentient beings.

## Figures and Tables

**Table 2:** Dire wolf de-extinction through five ethical frameworks.

Justification For De-Extinction	Underlying Ethical Principle	Key Critiques	Application to the Dire Wolf Case
Conservation tool	Utilitarianism (maximize future ecological benefit)	Could shift funding away from conserving living species [15] Does not address extinction causes [16] Not a true conservation tool [16,66] De-extinct species are not the true species [14,19]	Useful for recently extinct or extant relatives; less so for Pleistocene predators Dire wolves went extinct due to natural causes; reintroduction unlikely to support current conservation needs.
Ecological restoration	Environmental ethics (restore lost ecosystem functions)	Ecosystems have changed; roles may no longer exist or may cause harm [17]	Modern ecosystems lack a niche for the dire wolf; risk of ecological disruption
Scientific knowledge	Utilitarianism (advance science and technology)	Speculative results; all positive outcomes are speculative. Welfare risks for animals [16,17,39] created to serve human inquiry	Creating animals for knowledge when alternatives exist (e.g., modeling, comparative genomics).
Moral restitution	Deontological (duty to repair harm) Environmental ethics (restore lost ecosystem functions)	Dire wolves went extinct long before modern human influence. Justice and restoration arguments are flawed because it is not the same species [16,19,66].	Dire wolf extinction was not a human-driven extinction; “revived” individuals are not the same species.
Technological innovation	Virtue ethics (pursuit of human progress)	May prioritize novelty over responsibility or welfare May reflect hubris, human technological control over nature, ignoring moral humility [16]	This case seems to be more about novelty than necessity; undermines humility and responsibility in biotech
Public awe or species charisma	Relational ethics (emotional connection, existence value)	Can lead to Disneyfication of animals and nature [67]; can obscure animal interests De-extinction shifts our awe from nature itself to the admiration of human technology and control [15].	Is the coincidence with “Game of Thrones” hype really a coincidence? Public narrative can obscure welfare burdens and ethical complexity.

## Data Availability

No new data were created or analyzed in this study. Data sharing is not applicable to this article.

## Abbreviations

The following abbreviations are used in this manuscript:

GMO	Genetically Modified Organism
EU	European Union
CSO	Chief Science Officer
CITES	Convention on International Trade in Endangered Species of Wild Fauna and Flora
IUCN	International Union for Conservation of Nature
